# “Making the Mentally Ill Count”, lessons from a Health and Demographic Surveillance System for people with mental and neurological disorders in the Kintampo districts of Ghana

**DOI:** 10.1186/s13033-017-0130-x

**Published:** 2017-03-13

**Authors:** Kenneth A. Ae-Ngibise, Edward Adiibokah, Obed Ernest A. Nettey, Solomon Nyame, Victor Christian Korley Doku, Kwaku Poku Asante, Seth Owusu-Agyei

**Affiliations:** 1 Kintampo Health Research Centre, Ghana Health Service, Kintampo, Ghana; 2 Population Council, Accra, Ghana; 3 Centre for Global Mental Health, Institute of Psychiatry, Psychology and Neuroscience, King’s College London, London, UK

**Keywords:** Mental illness, Stigma and Exclusion, Enumeration exercise

## Abstract

**Background:**

Persons with mental and neurological disorders (PMNDs) are among the most marginalised groups in developing countries, as they are socially excluded and overlooked in most developmental efforts. Due to high levels of stigma and other operational difficulties, PMNDs are often marginalised in routine enumeration exercises. Health and Demographic Surveillance System is an important public health research platform especially in countries that lacks reliable data systems, as it registers and monitor basic demographic and health events such as births, deaths and migration in a geographically defined population. This information is essential for policy development and resource distribution and service delivery. We aim to document the reasons for not counting PMNDs in our communities and demonstrate the usefulness of the Kintampo Health and Demographic Surveillance Systems (KHDSS) platform in counting PMNDs over time. We also documented strategies in providing vital information that helps in establishing the rights of PMNDs.

**Methods:**

As a longitudinal study, psychiatric case register was established. Both quantitative and qualitative data collection techniques were used to solicit responses from stakeholders regarding the non-consideration of PMNDs as part of household membership in the study area. PMNDs were identified using the KHDSS and followed every 6 months. The “*targeted*” (actively searching for PMNDs) and “*service provision”* (providing medical treatment for PMNDs) approaches were adopted to enhance the identification of PMNDs.

**Results:**

Stigma was the main reason cited for the non-counting of PMNDs in the area. Following a “*targeted*” and “*service provision”* approach, the number of PMNDs enrolled into the psychiatric case register went up to 68% in 2010; as against the previous levels of 49 and 54% in 2005 and 2008 respectively. The study highlights the intrinsic value of such an approach for social inclusion of PMNDs.

**Conclusions:**

Stigma against PMNDs was report in this study. We provided evidence that the KHDSS platform is useful for identification of PMNDs for service provision. The paper highlights evidence for policy formulation and implementation.

## Background

Mental and Neurological conditions affect one out of every four individuals at some stage in life. These conditions include depression, substance use disorders, schizophrenia, epilepsy, Alzheimer’s disease, mental retardation and child and adolescence disorders [1]. These disorders have far–reaching public health and socio-economic consequences and the enormous contribution of mental illness to global disease burden is well documented [2, 3]. Mental disorders disproportionately affect the poor and other vulnerable groups and have deleterious effects on efforts at realising international development targets such as the Sustainable Development Goals [4, 5]. However, Persons with mental and neurological disorders (PMNDs) are among the most marginalised groups in low- and middle-income countries and they are forgotten in most development efforts [3]. Most national censuses in Africa includes people with mental disabilities but not specifically documenting PMNDs. The commitment of most governments and corporate bodies towards mental disorders is abysmal [6].

While some developed countries have embraced patient recovery-orientation as a guiding principle of their mental health policy, and thereby encouraging a partnership between mental health experts and users of mental health services [7], most nations in Africa have still yet to recognize the importance of PMNDs as these patients are still highly stigmatized [8, 9].

The prevalence of mental illness in Ghana is estimated to be approximately 13% of the adult Ghanaian population and about the same proportion among children, with a treatment gap of about 98% of the affected population [9, 10]. This translates to over 3 million people needing more attention in Ghana and as is the case in other African countries. It has also been predicted that there will be a projected increase in the number of young people entering the age at risk for onset of certain mental disorders [11, 12], thus worsening the current situation; yet there is low priority given to mental health service delivery in Ghana [13].

Stigmatisation of mental illness is a major problem affecting PMNDs and their relatives as well as institutions and health care providers for persons with mental illness [8, 9]. Therefore, PMNDs have lots of unmet needs, including maintaining their human dignity.

In order to address the specific needs of people with mental disorders, it is important to develop appropriate interventions that can be effectively evaluated. However, research evidence for policy formulation in most low- and middle-income countries is insufficient [14]. Basic and reliable epidemiological data on the prevalence and distribution of mental & neurological disorders is lacking in many low-income countries. Health and Demographic Surveillance Systems (HDSS) provide an invaluable platform for measuring health inequity and developing and evaluating health interventions [15]. The Kintampo Health Research Centre (KHRC) has since 2003 been operating a health and demographic surveillance system, referred to as the Kintampo Health and Demographic System (KHDSS), that identifies and follows up all residents in two adjoining districts in the middle belt of Ghana [16]. The database of residents is regularly updated to record births, deaths and migrations into and out of the KHDSS area. The KHDSS database also serves as a sampling frame for selecting risk-sets for participation in research studies.

Kintampo Health Research Centre established a mental health research unit In 2004 and has conducted a number of studies on neuro-psychiatric disorders that include; studies on psychosis [8, 17], mental disorders among older people, an epidemiology of postnatal depression [18–20] and the mental health and poverty project consortium which was carried in four African countries including Ghana, South Africa, Uganda and Zambia [17, 21–24]. These studies actively searched all households to identify potential cases of PMNDs and created a database of PMNDs referred to as “The Kintampo Psychiatric Case Register” (KPCR) that is being updated periodically.

In all of these studies, it was noticed that less than half (46 percent) of PMNDs living in the KHDSS area were registered in the KHDSS database. This study set out to investigate the ability to capture PMNDs as part of the KHDSS routine census updates in the communities.

## Methods

Both qualitative and quantitative data collection methods were used in this study.

### Study area

The Kintampo Health Research Centre (KHRC) in Kintampo North Municipality and Kintampo South District of Ghana in 2010 carried out the study. The study area with a resident population of about 140,000, is located in the Brong Ahafo Region of Ghana [16]. There are two government hospitals, two private hospitals, four health centres, one private clinic, 25 functional community health planning services zones and two private maternity homes in the two districts. Only one of the hospitals provides mental health services in the study area [8]. During the period of data collection, there were no social support programmes or counselling services for PMNDs. KHDSS is a member of The International Network of Health and Demographic Surveillance Sites (INDEPTH). The INDEPTH is a network of research institutions, which collects longitudinal data on demographic and health indices in defined geographical populations to inform policy and programme direction (http://www.indepth-network.org). In addition to population size dynamics, the KHDSS routinely collects information on socio-economic indicators such as household wealth, educational status and causes of death that are to provide extra information for analysing population-health inter-relationships for policy.

### The qualitative approach

The qualitative approach involved in-depth interviews (IDIs) and participant observation. These methods were employed to provide an understanding of the context, processes and reasons for initial non-registration of PMNDs in the KHDSS area and to describe strategic responses that were adopted by the mental health unit of KHRC to encourage counting of PMNDs as part of the census update. Sampling of respondents for IDIs was purposive and issues were tailored to suit each subgroup. An interview guide was developed that focused on documenting the reasons for low registration of PMNDs. Interviews were conducted by trained research officers. Fifteen IDIs were conducted among 10 heads of households with a mentally ill person and 5 field staff of the KHDSS. The age range of the household heads was 30–65 years and that for the fieldworkers was 23–30 years. Half of the household heads were males while all the fieldworkers were males. The local language (Twi) was used to conduct interviews with heads of households with a PMND. These interviews were back translated into English for the analysis. Interviews were recorded and transcribed verbatim. Guided by objectives of the study, content analysis [25, 26] was used to search for appropriate themes. QSR NVivo [27] version 8 software was used to categorise the data into themes and to discern patterns emerging from the themes. Participant observation was also used to supplement information gathered from the in-depth interviews. The authors were deeply involved in activities of the various stakeholders including work of KHDSS, service providers and Kintampo Multi-Sectorial Forum (this forum was established to act as a platform for non-hierarchical interaction between stakeholders in mental health service delivery including PMNDs). The PMNDs were not included as respondents for this study because our main aim was to get the perspective of stakeholders (including family heads of PMNDs) on why they routinely excluded PMNDs from their family membership register. There were also visits to 13 traditional and faith healing centres, where observations were made about how PMNDs were being catered for.

### The quantitative approach

This was a longitudinal follow-up study of PMNDs between 2004 and 2010. A longitudinal psychiatric case register referred to as the KPCR was established with the main aim of linking PMNDs to health care. Information captured in the KPCR includes the KHDSS unique identification number as well as basic demographic characteristics such as age, gender, education, health insurance cover, diagnosis and others. These cases were initially identified through the baseline mental health study in 2004, which assessed risk factors for schizophrenia and related psychosis. This matched case control study was the first population based conducted in the region to test the hypothesis that cannabis use is a risk factor for the onset and development of schizophrenia. Subsequently, there were other mental health studies including the Mental Health and Poverty Project, care givers burden study and Studies on the Epidemiology of Epilepsy in Demographic Surveillance Sites [8, 28, 29] that provided the opportunity to update the case register. These identified cases were linked to psychiatric services in 2008 when the first community psychiatric nurse was posted to the district. The database of cases that were established from these studies were often cross-checked with the KHDSS database, which serves as the master database of all residents in the study area. The purpose was to ascertain whether or not PMNDs are actually being counted as members in their households through KHDSS routine census updates. The linkage was done through each individual’s household identification number and other unique identifiers. We present the percentage distribution of the cases identified during the period.

### Strategies adopted to improve the identification of PMNDs

**Fig. 1:**
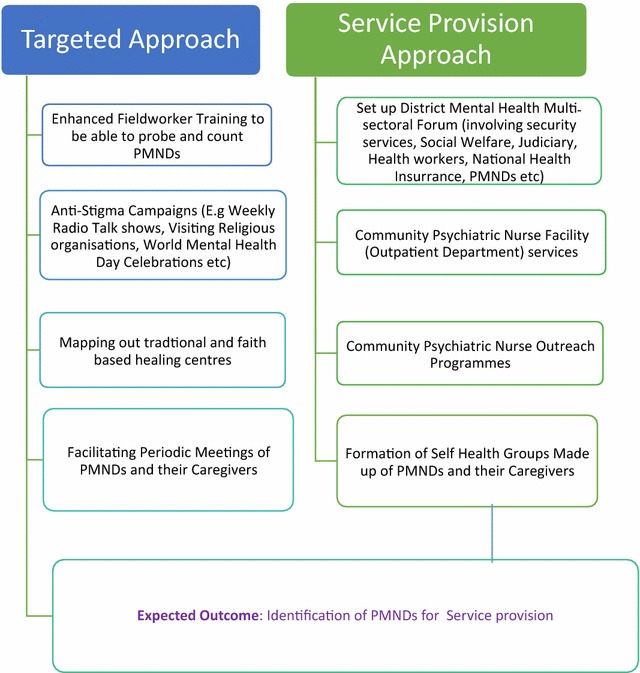
Strategies adopted to get PMNDs counted in society

There were two main strategies which were adopted to improve the identification of PMNDs. These were the targeted and service provision. The targeted approach was aimed at improving the ability of the KHDSS to be able to identify PMNDs. The service provision method involved the introduction of mental health services in Kintampo district where no previous services existed. These approaches are presented in Fig. 1.

## Results

Results of this study are presented in four subsections. The first section gives a summary of the socio-demographic characteristics of PMNDs in the study area. The second section, presents the factors responsible for the initial low coverage of PMNDs in the KHDSS. This is followed by the strategies that were adopted to ensure the inclusion of PMNDs in the KHDSS, and value of the HDSS platform for longitudinal studies and interventions for PMNDs.

### Socio-demographic characteristics and classifications of PMNDs in the Kintampo districts

Table 1 shows the socio-demographic characteristics of PMNDs in the Kintampo area, using the 2010 KPCR. There were slightly more females compared with males. Notably, more than 65% of cases did not have a health insurance cover.

**Table 1:** Demographics and classifications of PMNDs in Kintampo in 2010

Variable	Population	Percentage
Case type		
Mental disorders	329	57.8
Epilepsy	240	42.2
Gender		
Male	261	45.9
Female	308	54.1
Age group		
<18	135	23.7
18–34	223	39.2
35–64	175	30.8
65+	34	6.0
Education		
None	279	49.0
Basic	228	40.1
Secondary	30	5.3
Tertiary	11	1.9
Vocational	21	3.7
Marital status		
Single	377	66.3
Married	135	23.7
Cohabiting	31	5.4
Widowed	26	4.6
National Health Insurance scheme		
Registered	198	34.8
Unregistered	369	65.2

### Factors responsible for initial low registration of PMNDs in the KHDSS

The results of semi-structured interviews revealed that though household heads were required to provide information on all members of their households in the periodic census and vital events updates as part of the KHDSS, some of them declined to mention members of their households with mental disorders. As depicted in the dialogue below, family members with mental illness were not regarded as part of their families and therefore not worthy to be counted.

Question… why is it that you never mention Amadu as a member of your household when we come here?

Answer:..….. “Anytime, you come and we are talking about people you insist on talking about Amadu.” (IDI, Household Head).

The responses from the fieldworkers also corroborated this assertion:

“Some of them (household members) always feel shy….when you go to the household, they don’t remember to add their (referring to person with mental disorder) names as household members, they don’t even count them as human beings”, (IDI, Field Worker of KHDSS).

Inadvertently, some field workers who are community outreach agents of the KHDSS also harboured negative mental attitudes towards PMNDs and therefore failed to probe for the inclusion of any persons with mental disorders. This situation was succinctly captured in the extract below:

“They (referring to fieldworkers) have a negative attitude towards these people with mental illness and therefore do not care if they are included or not” (IDI, staff of KHDSS).

Other reasons given for the over 50% non-inclusion of PMNDs in the initial KHDSS database were stigma and the use of pseudo-names (aliases). These two reasons run through most of the interviews with staff of the KHDSS. They indicated that some of the names of PMNDs in the KPCR were not the same names they bore in the community and hence in the KHDSS database. This made it difficult for them to be traced when the KHDSS went to visit on their regular updates. A respondent in an interview said;

“One of the main reasons accounting for these discrepancies in coverage of PMNDs is the fact that PMNDs have several names and hence depending on who provides the information on household membership to census fieldworkers, this problem will continue to persist”, (IDI Staff of KHDSS).

### Strategic responses adopted to get PMNDs counted

A number of strategies were adopted to ensure that PMNDs were counted as presented in Fig. 1. These involved two interlinked strategies, the “targeted approach” and “service provision approach”. The “targeted approach” was primarily geared towards enhancing ability of the KHDSS to access PMNDs who are normally hidden and hard to reach. There was an enhanced field worker training to include identification and referral of potential mentally ill patients. Notably the fieldworker training addressed issues of fieldworker’s attitude towards PMNDs and probing skills to illicit information about PMNDs. The wider community was also targeted through anti-stigma campaigns using community radio, talk shows among various religious bodies and other educational activities on special days such as World Mental Health Day. An important component of this targeted approach was involvement of PMNDs in anti-stigma activities. This included periodic scheduled meetings of users and their caregivers of PMNDs to discuss matters of common interest.

The second approach was a strategic response to service provision with the aim of improving mental health service in a district where there was no existing formal mental health service. The approach involved three interlocking components: One was setting up a District Mental Health Multi-Sectorial Forum described earlier; this forum includes PMNDs aimed at committing service providers and other identified stakeholders towards the plight of PMNDs and what roles they are expected to play in order to help PMNDs. Another component was the Community Psychiatric Nurse (CPN) led clinical services (including periodic community outreach programmes), aimed at providing treatment to PMNDs at the community level through a task-shifting strategy; general health workers were trained on recognition and management of mental disorders and the need to collaborate with traditional and faith-based healers. In recognition of the role of social determinants of managing mental illness, a final component involved the formation of self help groups made up of PMNDs and their caregivers.

**Fig. 2:**
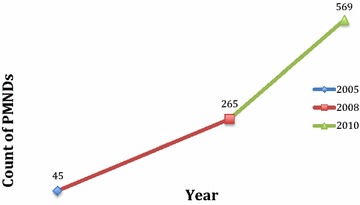
Number of cases enumerated by KHDSS from 2004 to 2010. *NB* the numbers over time include new cases and those who were not previously counted

These strategies were beneficial in two ways. Apart from the aim of improving ability of KHDSS to cover PMNDs, the strategies also proved invaluable in linking PMNDs to mental health services and other interventions. As depicted in Fig. 2, the number of cases captured by the KHDSS increased significantly demonstrating importance of KHDSS in tracking PMNDs through routine census updates.

## Discussion

This study set out to investigate factors that led to not counting people with neuro-psychiatric conditions as part of the resident population of the Kintampo district as prescribed by the Kintampo Health and Demographic Surveillance System (KHDSS), come up with strategies that can be adopted to integrate PMNDs whiles highlighting such strategies in the KHDSS platform for longitudinal studies and interventions involving PMNDs and others living with stigmatizing conditions.

The study results highlight a higher proportion of females compared with males have mental disorders in the study area. This is comparable with a study on the experience of caregivers of mental patients in the study area that reported a higher proportion of females suffering from mental disorders than males [8]. There are other reports in Africa which suggest that mental illness is common in females compared to males [30] and also in other parts of the world [31].

Persons with mental and neurological disorders are supposed to be registered free of charge by the national health insurance scheme, however, more than 65% of the mental health cases did not have a health insurance cover. A system that captures basic data of all members of the communities, including those with mental and neurologic disorders is needed to ensure all deserving people are registered with the national health insurance scheme. As a result of this study, there were discussions with the municipal health insurance scheme authority, and it was agreed that insurance cover will be provided for people identified as living with mental and neurological disorders.

Stigma reduction was directly targeted in the strategic responses, as it was cited as the main reason for PMNDs not being counted in the main enumeration and update exercises of the KHDSS. This is not surprising, as stigma has been known to be the main cause of social exclusion for people with mental illness. Earlier studies in mental health in the area indicated that stigma and discrimination against people with mental illness was very high and deeply ingrained in the socio-cultural practices of the people [17]. This finding is not atypical of what pertains in other African countries, as a survey in Nigeria reported over 80% of participants saying that they would be ashamed if people knew someone in their family as having a mental illness [32]. In the national surveys or census in Ghana, counting of PMNDs is low as they are grouped with other people with physical disabilities [33]. We conjecture here that this situation depicted a subtle attempt by members of families to distance themselves from the PMNDs and protect the family from any stigma associated with a family member perceived as having a mental illness. All these narratives point to the fact that PMNDs are often excluded, are hard to reach and need special efforts to get them counted in the routine enumeration exercises.

### Importance of the KHDSS

The KHDSS platform also has a fundamental value for the inclusion of PMNDs and others living with stigmatizing conditions in research and other social interventions. After the introduction of strategic interventions, there was a consistent yearly increase in identification of PMNDs in the KHDSS. The cases increased to 569 and 387 on the PCR and KHDSS respectively in 2010 as a result of the strategic interventions adopted. The benefits of KHDSS platform can be classified into two broad categories. The first is its value in locating and linking PMNDs who were hitherto unavailable and hard to reach, to services and interventions. The second is its value in supporting longitudinal studies in mental health. It is important to note that there is the need for special surveys to identify PMNDs as its being done for other diseases.

A very important contribution of the KHDSS was the creation of the KPCR. The KPCR described previously is longitudinal database of people with psychiatric disorders, which records basic demographic characteristics and classifications of PMNDs in the Kintampo North and South Districts of Brong Ahafo Region of Ghana. The KPCR is used to describe the patterns of current and future referrals to mental health services, to describe the epidemiology of mental and neurological disorders and serves as the basis for a district-mental health management information system [34]. Functioning of the case register is facilitated by its linkage to the KHDSS, which is the primary source of basic information on cases to start with and regular or periodic updates. Although cases and their caregivers were first identified and referred by trained fieldworkers and Community Key Informants of the KHDSS, the diagnostic category of cases is usually confirmed by a CPN. The CPN uses the International Classification of Diseases 10th Edition, primary care version for mental disorders (ICD-10, PCV) for clinical diagnosis.

The KHDSS platform was also very useful in the services provision approach. First the unique identifier provided by the KHDSS and the fact that PMNDs are situated within households also facilitates the home and or community visits by the CPNs for service delivery. With provision of compound numbers as well as personal identification numbers for all residents in the district which includes these cases, the KHDSS makes it possible for the cases to be traced to their houses for further detailed assessment in the routine home visits of the CPN.

Another important feature of the KHDSS was its value in mapping out traditional and faith healer systems. A salient issue that emerged from the study was that most of the PMNDs were hidden in traditional healer centres and prayer camps. In our visits to the thirteen existing prayer camps/traditional healer centres, we came across some PMNDs who were staying in camps/centres with their relatives, while others had been abandoned. Traditional and faith healers were viewed as the de-facto source of treatment for the mentally ill (personal conversation with faith based healers). Therefore, the KHDSS system was used to map out the various traditional and faith healer centres in the catchment area using a geographical information system (GIS). This mapping system proved very useful in linking the services offered by the traditional and faith based healers with the CPN services. This complementary mental health services seems to be accepted by the mental health patients and their caregivers.

The KHDSS database was also extremely helpful in locating and including PMNDs in government-sponsored social protection schemes (being registered as indigents by the National Health Insurance Scheme) for the vulnerable under the Mental Health and Poverty Project [29]. This is especially important in a context where people with mental illness are mostly excluded from social protection schemes for the vulnerable. The KHDSS platform was also useful in supporting other mental health studies. It is now feasible to undertake Epidemiological and other large-scale studies on mental and neurological disorders. For instance, the Studies of the Epidemiology of Epilepsy in Demographic Sites (SEEDS) initiative; an INDEPTH network multi-country, multi-site epilepsy prevalence, causes and outcome study, was successfully carried out using the KHDSS platform. Prevalence and socio-cultural determinants of dementia in Ghana also used the KHDSS platform to recruit study participants. Several proposed studies are earmarked using the KHDSS database as the platform; these include: excess mortality among PMNDs, mental health and malaria study, adolescent mental health studies, depression and HIV/AIDS. This platform will ensure optimal design and implementation.

## Conclusion

People with mental and neurological disorders suffer from social exclusion because of perceived stigma. Getting them counted is a first step towards addressing their numerous needs. The KHDSS has provided an invaluable platform of longitudinal database for studying social determinants of mental and neurological disorders. Mental health services provision is one of the ways of addressing stigma and the marginalization against PMNDs. The services approach adopted by KHDSS, offers an important gateway towards providing recovery, addressing stigma and ensuring that they are counted. Mainstream enumeration exercises such as national censuses could take a cue from this study by specifically targeting PMNDs who are normally invisible and hard to reach.

## References

[1] WHO. Fact Sheet: The World Health Report 2001. Geneva; 2001.

[2] Group, L.G.M.H. (2007). Scale up services for mental disorders: a call for action. Lancet.

[3] Whiteford HA (2013). Global burden of disease attributable to mental and substance use disorders: findings from the Global Burden of Disease Study 2010. Lancet.

[4] Alleyne G (2013). Embedding non-communicable diseases in the post-2015 development agenda. Lancet.

[5] Skeen S (2010). ‘Mental health is everybody’s business’: roles for an intersectoral approach in South Africa. Int Rev Psychiatry.

[6] Murray CJ, Lopez AD (1996). Evidence-based health policy—lessons from the Global Burden of Disease Study. Science.

[7] Amering M, Schmolke M (2009). Recovery in mental health: reshaping scientific and clinical responsibilities.

[8] Ae-Ngibise KA (2015). The experience of caregivers of people living with serious mental disorders: a study from rural Ghana.

[9] Barke A, Nyarko S, Klecha D (2011). The stigma of mental illness in Southern Ghana: attitudes of the urban population and patients’ views. Soc Psychiatry Psychiatr Epidemiol.

[10] WHO. Ghana: a very progressive mental health law. The country summary series. 2007. http://www.who.int/mental_health/policy/country/GhanaCoutrySummary_Oct2007.pdf.

[11] Gaisie S, Gyau-Boakye P. Population growth, water/sanitation and health. Population, health and development in Ghana: attaining the Millennium Development Goals; 2007. pp. 91–134.

[12] Flisher AJ (2007). Mental health policy development and implementation in four African countries. J Health Psychol.

[13] Doku V (2011). Stakeholders’ perceptions of the main challenges facing Ghana’s mental health care system: a qualitative analysis. Int J Cult Ment Health.

[14] Saraceno B (2007). Barriers to improvement of mental health services in low-income and middle-income countries. Lancet.

[15] Mwageni E (2005). Socio-economic status and health inequalities in rural Tanzania: evidence from the Rufiji demographic surveillance system. Measuring health equity in small areas—findings from demographic surveillance systems.

[16] Owusu-Agyei S (2012). Demographic patterns and trends in Central Ghana: baseline indicators from the Kintampo Health and Demographic Surveillance System. Glob Health Action.

[17] Read UM, Adiibokah E, Nyame S (2009). Local suffering and the global discourse of mental health and human rights: an ethnographic study of responses to mental illness in rural Ghana. Global Health.

[18] Weobong B (2014). Prevalence and determinants of antenatal depression among pregnant women in a predominantly rural population in Ghana: the DON population-based study. J Affect Disord.

[19] Weobong B (2015). Determinants of postnatal depression in rural Ghana: findings from the Don population based cohort study. Depression Anxiety.

[20] Weobong B (2009). The comparative validity of screening scales for postnatal common mental disorder in Kintampo, Ghana. J Affect Disord.

[21] Ae-Ngibise K (2010). ‘Whether you like it or not people with mental problems are going to go to them’: a qualitative exploration into the widespread use of traditional and faith healers in the provision of mental health care in Ghana. Int Rev Psychiatry.

[22] Read U (2012). “I want the one that will heal me completely so it won’t come back again”: the limits of antipsychotic medication in rural Ghana. Transcult Psychiatry.

[23] Read UM, Doku V (2013). Mental health research in Ghana: a literature review. Ghana Med J.

[24] Awenva AD (2010). From mental health policy development in Ghana to implementation: what are the barriers?. Afr J Psychiatry (Johannesbg).

[25] Elo S, Kyngäs H (2008). The qualitative content analysis process. J Adv Nurs.

[26] Hsieh H-F, Shannon SE (2005). Three approaches to qualitative content analysis. Qual Health Res.

[27] Hutchison AJ, Johnston LH, Breckon JD (2010). Using QSR-NVivo to facilitate the development of a grounded theory project: an account of a worked example. Int J Soc Res Methodol.

[28] Doku V, et al. Phase 1. Country report: a situation analysis of mental health policy development and implementation in Ghana. Phase 1. Country report: a situation analysis of mental health policy development and implementation in Ghana; 2008.

[29] Ae-Ngibise KA, et al. Prevalence and risk factors for Active Convulsive Epilepsy in Kintampo, Ghana. Pan Afr Med J. 2015;21(29). doi:10.11604/pamj.2015.21.29.6084. 10.11604/pamj.2015.21.29.6084 PMC4561141 26401223

[30] Herman AA (2009). The South African Stress and Health (SASH) study: 12-month and lifetime prevalence of common mental disorders. SAMJ South Afr Med J.

[31] Seedat S (2009). Cross-national associations between gender and mental disorders in the World Health Organization World Mental Health Surveys. Arch Gen Psychiatry.

[32] Gureje O (2005). Community knowledge of and attitude to mental illness in Nigeria. Br J Psychiatry.

[33] GSS. Ghana Statistical Service: 2010 Population and Housing Census (PHC). 2012.

[34] Knatterud GL (1965). A psychiatric case register for mental health planning. Public Health Rep.

